# Prevalence and associated Risk Factors of Severe Early Childhood Caries in 12- to 36-month-old Children of Sirmaur District, Himachal Pradesh, India

**DOI:** 10.5005/jp-journals-10005-1431

**Published:** 2017-06-01

**Authors:** Ritu G Mangla, Raman Kapur, Abhishek Dhindsa, Manish Madan

**Affiliations:** 1 Redaer, Department of Pedodontics and Preventive Dentistry, Himachal Institute of Dental Sciences, Paonta Sahib, Himachal Pradesh India; 2 Professor, Department of Pedodontics and Preventive Dentistry, Swami Devi Dyal Hospital and Dental College, Panchkula, Haryana India; 3 Professor, Department of Pedodontics and Preventive Dentistry, Maharishi Markandeshwar College of Dental Sciences and Research Ambala, Haryana, India; 4 Professor and Head, Department of Pedodontics and Preventive Dentistry, Himachal Institute of Dental Sciences, Paonta Sahib, Himachal Pradesh India

**Keywords:** Prevalence, Risk factors, Severe early childhood caries.

## Abstract

**Aim:**

To assess the prevalence, distribution, and associated risk factors of severe early childhood caries (S-ECC) among 12- to 36-month-old children of district Sirmaur, Himachal Pradesh, India.

**Materials and methods:**

The present study was conducted on a random sample of 510 children, both boys and girls, between 12 and 36 months of age randomly selected from various government-sponsored day-care centers, private day-care centers, and vaccination centers. Caries was recorded using World Health Organization criteria. Statistical analysis was done by using chi-square test and Mann-Whitney test. A two-sided p value was calculated for each statistical test. Multiple logistic regressions were done to calculate the risk of S-ECC from independent variables.

**Results:**

In the present study, S-ECC was found in 21% of 510, 12 to 36 months old children of Sirmaur district, Himachal Pradesh. The S-ECC was found to be significantly higher in 25 to 36 months old children’s age group and was 27.8% in them as compared with 8% in 12 to 24 months old children.

**Conclusion:**

Providing anticipatory guidance and education to parents is essential for the promotion of optimal oral health of their children. There is a need for moving upstream to propose and implement policies and programs to improve the oral health of the very young, especially in a developing country like India, which lacks much data on S-ECC.

**How to cite this article:**

Mangla RG, Kapur R, Dhindsa A, Madan M. Prevalence and associated Risk Factors of Severe Early Childhood Caries in 12- to 36-month-old Children of Sirmaur District, Himachal Pradesh, India. Int J Clin Pediatr Dent 2017;10(2):183-187.

## INTRODUCTION

Early childhood caries (ECC), a term suggested at a 1994 Centers for Disease Control and Prevention workshop, denotes any form of caries occurring in the primary dentition of infants and youngsters.^1^

The disease of ECC is defined as “the presence of 1 or more decayed (noncavitated or cavitated lesions), missing (due to caries), or filled tooth surfaces“ in any primary tooth in a child 71 months of age or younger. In children younger than 3 years of age, any sign of smooth surface caries is indicative of severe early childhood caries (S-ECC). From ages 3 through 5, one or more cavitated, missing (due to caries), or filled smooth surfaces in primary maxillary anterior teeth, or a decayed, missing, or filled score of >4 (age 3), >5 (age 4), and >6 (age 5) surfaces constitutes S-ECC.^2^

Severe early childhood caries is a specific form of rampant decay of primary teeth in infants. The lesions develop quickly and occur in surfaces generally considered to be at low risk for caries. This caries pattern is observed in late infancy and early childhood.^3^ In contrast to the extensive reporting of the changing patterns of dental caries in the permanent dentition, there are fewer reports on trends in dental caries in primary teeth, particularly in preschool children under 3 years of age.^4^

Children who have caries in their primary teeth in infancy or as toddlers tend to develop additional dental decay in their primary teeth and are also more likely to develop dental caries in their permanent dentition. Therefore, the most important target group for instituting preventive programs seems to be infants and toddlers.^5^

Sirmaur is the most southeastern district of Himachal Pradesh, India. It has an area of 2,825 km^2^ and a population of 5.3 lakh.^6^

Focusing on 12- to 36-month-old children attending government-sponsored day-care centers, private day-care centers, and vaccination centers in Sirmaur district of Himachal Pradesh, the present study aimed to identify occurrence, distribution, and associated risk factors of S-ECC in 12- to 36-month-old children of district Sirmaur, Himachal Pradesh.^7^

## MATERIALS AND METHODS

The present study was conducted on a random sample of 510 children, both male and female, between 12 and 36 months of age. The children were randomly selected from various government-sponsored day-care centers, private day-care centers, and vaccination centers from southeast part of Sirmaur district, Himachal Pradesh. The proposed study was reviewed by the ethical committee of Maharishi Markandeshwar University and clearance was obtained for the same.

Prior to the visit, written official permission to conduct the study was taken from all the concerned authorities as under:

- Child Development Project Officer for government-sponsored day-care centers.
- Principal/in-charge of various private day-care centers.
- Chief Medical Officer of Sirmaur for various vaccination centers.
- Parents/caretaker of the child.

Prior information was provided through in-charge of respective centers to the caregivers of the children attending various government-sponsored and private day-care centers regarding the dental examination of their child and the interview of the caregiver to be conducted. At the vaccination centers, the caregiver of the children attending these centers was informed of the nature of investigation, and prior permission and informed written consent of parents or caretakers were taken at the respective centers.

In this study, the required data were collected and recorded using printed proformas, which consisted of name of the child, age, sex, address, phone number, parent’s education, occupation locality and included questions about child’s dietary, feeding, oral hygiene habits, illnesses, etc.

Oral hygiene practices (use of toothbrush, materials used, and frequency of brushing) were also recorded. This proforma was filled by the examiner.

Clinical examinations were carried out at the immunization center’s medical room or classroom at the day-care centers with the aid of a mouth mirror, Community Periodontal Index (CPI) probe, and under natural light or where required with the aid of battery-operated head light/torch light.

The subjects were examined on an upright chair. Disposable mouth masks and gloves were used during the examination. The subjects were examined using an autoclaved plain mouth mirror and CPI probe. Caries was recorded as per World Health Organization criteria.^7^

### Data Analysis

Data were entered into a database, checked for errors, and analyzed by Statistical Package for the Social Sciences software (release 6.1 version). Statistical analysis was done using chi-square test and Mann-Whitney test. A two-sided p value was calculated for each statistical test. We applied multiple logistic regression to calculate the risk of S-ECC from independent variables.

## RESULTS

**Graph 1:**
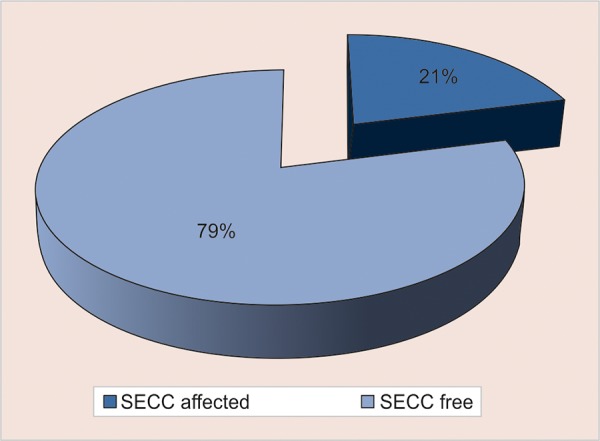
Prevalence of S-ECC in 12 to 36 months old children in Sirmaur district, Himachal Pradesh

**Graph 2:**
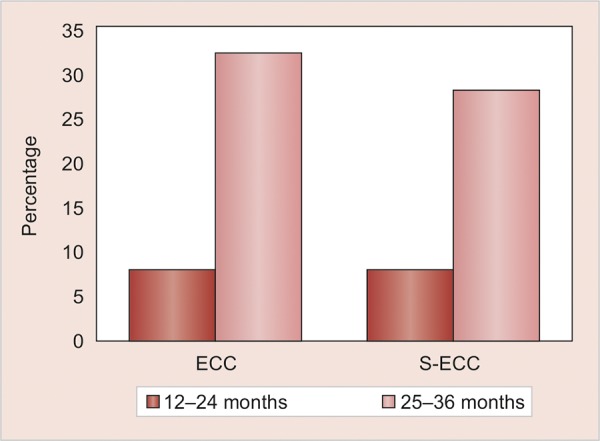
Occurrence of ECC and S-ECC according to age groups

Out of 510 [220 females (43.18%) and 290 (56.86%) males] 12- to 36-month-old children examined, 175 (34.3%) children were in the age group of 12 to 24 months and 335 (65.7%) were in the age group of 25 to 36 months. In the present study, S-ECC was found in 21% of 510, 12 to 36 months old children of Sirmaur district, Himachal Pradesh (Graph 1). The S-ECC was found to be significantly higher in 25 to 36 months old children’s age group and was 27.8% in them as compared with 8% in 12 to 24 months old children (Table 1, Graph 2).

**Table 1:** **Table 1:** Occurrence of ECC and S-ECC according to age groups

				*ECC*				*S-ECC*			
*Age groups*		*Total number* *of children (%)*		*N*		*%*		*N*		*%*	
12-24 months		175 (34.3)		14		8		14		8	
25-36 months		335 (65.7)		105		32		93		27.8	
Total		510		119 (23.3)		23.3		107		21	
		p-value χ^2^		0.000*		37.673		0.000*		27.076	

*Stands for statistically significant as p ≤ 0.005

Mean decayed, filled teeth (dft) scores in the study population was found to be 1.01 ± 2.373. In 12 to 24 months old children, mean dft scores were found to be 0.25 ± 1.030 and mean dft scores in 25 to 36 months old children were found to be 1.41 ± 0.750; mean decayed, filled surfaces (dfs) score in the study population was found to be 1.36 ± 3.998. Mean dfs score for 12 to 24 months old children in the study population was found to be 0.47 ± 2.286. Mean dfs score for 24 to 36 months old children in the study population was found to be 1.83 ± 4.583 (Table 2).

**Table 2:** **Table 2:** Age groupwise distribution of mean dft and mean dfs in 510 children

*Age group* *(months)*		*N*		*Mean dft ± Std.* *deviation*		*Mean dfs ± Std.* *deviation*	
12-24		175		0.25 ± 1.030		0.47 ± 2.286	
25-36		335		1.41 ± 0.750		1.83 ± 4.583	
Total		510		1.01 ± 2.373		1.36 ± 3.998	

**Table 3:** **Table 3:** Factors related to S-ECC explained by logistic regression model

						*95% confidence interval*			
*S-ECC logistic regression model Variables*		*Significance*		*Odds ratio*		*Lower*		*Upper*	
Age group		<.0001**		7.059		3.219		15.476	
Mother working or nonworking		0.312		1.401		0.729		2.693	
Income		0.494		1.049		0.914		1.204	
Mother’s education level		0.001**		0.571		0.405		0.806	
Father’s education level		0.083		1.347		0.962		1.886	
Socioeconomic status		0.885		1.042		0.595		1.825	
Bottle-feeding done on demand		0.510		2.347		0.186		29.686	
Bottle sipping during the day		0.237		4.043		0.399		40.937	
Bottle fed to sleep		0.702		1.901		0.071		50.781	
Duration of bottle-feeding		0.018*		0.508		0.289		0.892	
Contents of bottle-feeding		0.992		0.998		0.712		1.399	
Any sugar added to milk		0.289		1.609		0.668		3.879	
Age of commencement of solids		0.873		0.964		0.617		1.508	
Current adult supervision of toothbrushing		0.003**		0.367		0.189		0.710	
Frequency of consumption of sweet and sticky food		0.010*		1.552		1.110		2.170	
Whether sweets are given as reward to the child		0.737		0.833		0.286		2.423	
Constant		0.000		0.011					

*Stands for statistically significant as p ≤0.005; **Stands for statistically highly significant as p ≤ 0.001

Multivariate analysis was carried out using a stepwise multiple logistic regression to determine the strongest factors that were independently related to S-ECC when other variables were held constant. All variables were included in the start and those failing to show a significant relationship to S-ECC were subsequently removed in a step-wise manner. After logistic regression, which analyzes the effect of each factor when controlled across all other factors, many of the early significant relationships disappeared (Table 3).

The factors remaining statistically significant were:

- Age group of the child
- Mother’s level of education
- Duration of bottle-feeding
- Current adult supervision of toothbrushing
- Frequency of consumption of sweet and sticky foods

Mothers educated up to high school level showed higher percentage of children with S-ECC.

About 60.7% (65) children with S-ECC were bottle-fed up to 13 to 36 months. Thus, more number of children with S-ECC were bottle-fed for prolonged duration.

In 37.4% (40) children with S-ECC, adults supervised their toothbrushing and in 62.6% (67) children, adults did not supervise their toothbrushing.

In 31.8% (34) children, frequency of consumption of sweet and sticky food was twice. Thus, more the number of times sweet and sticky food is consumed by children, more are the children affected by S-ECC.

## DISCUSSION

The measurement of oral health is important for understanding normal biological processes and the natural history of disease and for planning and evaluating health services.^8^ India is a developing country, facing many challenges in rendering oral health care to masses. Dental caries is highly prevalent in India, which is influenced by the lack of awareness among the public. There is a dearth of awareness and information about the oral health of preschool children in India.^9^

In the present study, S-ECC was found in 21% of 510, 12 to 36 months old children of Sirmaur district, Himachal Pradesh. The mean dft score was 1.01 ± 2.373 and the mean dfs score was found to be 1.36 ± 3.998. Azevedo et al^3^ in 2005 reported S-ECC in 36% of the 36 to 71 months old children examined. Jin et al^10^ found S-ECC in 47% of children aged 6 to 59 months with a mean decayed, missing, filled teeth (dmft) score of 1.93 ± 0.27. The difference in the present study may be due to the inclusion of higher age groups in other studies.

The multivariate logistic regression model showed that older age of the child was a major risk factor that affects S-ECC. The severity of caries, as reflected by mean dft and dfs, was seen to increase with age. Chi-square test reveals that a significant correlation exists between the mean dft and age of children. Similar increase in caries experience has been found by other investigators.^11-14^ This may be explained by the fact that if caries-inducing factors are persistently present in affected children, the accumulative effect results in an increase in the severity of caries with age.^15^

Also, mother’s low level of education was found to be an important risk factor. In this study, children with S-ECC belonged to more of mothers with low level of education up to high school or primary school. The difference between S-ECC experience among children with regard to mother’s educational level could be attributed to lack of knowledge and awareness of mothers regarding dental health, oral hygiene practices, and feeding habits. Mothers completing higher level of education are more informed in health questions, and these influence various behaviors related to health as to maintain good dietary and hygiene behaviours.^16^ Similar results have been reported in most studies.^15,17-19^

In addition, S-ECC was also more likely to occur if the toothbrushing was not supervised as there were significantly more children with S-ECC in whom the brushing was not supervised and they brushed alone rather than being assisted. Similar findings were observed by Mahejabeen et al.^20^

Prolonged duration of bottle-feeding was the next major contributing risk factor for S-ECC. Bottle-fed children in this study were more likely to have S-ECC than those whose caregivers reported that they did not receive a bottle. However, prolonged duration of bottle-feeding was found to be a major risk factor in S-ECC, according to the multiple regression analysis. Nonetheless, mothers and other caregivers should be informed of how they can reduce the probability of S-ECC by avoiding inappropriate bottle use and contents, discouraging breastfeeding on demand, transitioning to the use of a regular cup at 12 months of age, and cleaning the child’s mouth regularly, once the first primary tooth has erupted. Improper feeding habits were found to be important attributive factors for S-ECC.

Severe early childhood caries was found to be higher in children consuming sweet and sticky snacks more frequently. Statistically significant positive correlations were found between S-ECC and frequency of consumption of sweet and sticky snacks in other studies also.^17,21,22^

This calls for improvement in children’s oral health maintenance among all those who serve as dominant primary caregivers of the children. There is a need to inform parents about necessary prevention, education, and dental care at early stages of development. Oral health promotion programs should be extended to all health care facilities where children from all socioeconomic levels are visiting from infancy onward.

Parents of children in India usually do not realize the importance of primary dentition considering that permanent teeth would soon erupt even if the primary teeth are decayed. Their lack of knowledge about the importance of oral health of their wards is a contributory factor for oral health decline in the very young.

The early caries development seen in these young children reinforces the need for early caries examinations and initiation of prevention programs before, or soon after, tooth eruption.

## CONCLUSION

Occurrence of S-ECC was found to be 21% among 12 to 36 months old children of Sirmaur district, Himachal Pradesh, with a mean dft score of 1.01 ± 2.373 and mean dfs score of 1.36 ± 3.998. Evaluating the relationship of S-ECC with various associated risk factors, the factors found to have highly significant correlation value, in decreasing order were as follows:

- Age group (mean dft score in 25-36 months age group was found to 1.41 ± 0.750 and mean dft score of 0.25 ± 1.030 in 12-24 months old children)
- Mother’s low level of education
- Prolonged duration of bottle-feeding
- Current adult supervision of toothbrushing.
- Frequency of consumption of sweet and sticky food

Providing anticipatory guidance and education to parents is essential for the promotion of optimal oral health of their children. Young children constitute a vulnerable population because of their dependence and inability to communicate their needs. Oral health disparities continue to pose challenges as dental caries is the most common chronic disease of childhood. Therefore, there is a need for moving upstream to propose and implement policies and programs to improve the oral health of the very young, especially in a developing country like India, which lacks much data on S-ECC.
